# Early detection of delayed pneumothorax using lung ultrasound after transthoracic needle lung biopsy: A prospective pilot study

**DOI:** 10.1111/crj.13495

**Published:** 2022-05-20

**Authors:** Jeong Suk Koh, Chaeuk Chung, Ju Ock Kim, Sung Soo Jung, Hee Sun Park, Jeong Eun Lee, Da Hyun Kang, Yoonjoo Kim, Dongil Park

**Affiliations:** ^1^ Division of Pulmonary and Critical Care Medicine, Department of Internal Medicine Chungnam National University Hospital Daejeon Republic of Korea

**Keywords:** delayed pneumothorax, lung sliding, lung ultrasound, pneumothorax, transthoracic needle lung biopsy

## Abstract

**Objectives:**

Delayed pneumothorax can cause an emergency room visit and be life‐threatening in case of tension pneumothorax after transthoracic needle biopsy. We hypothesized that most delayed pneumothoraces are diagnosed by later enlargement of occult pneumothorax due to the low diagnostic accuracy of a chest X‐ray. Lung ultrasound is a highly accurate tool for detection of pneumothorax. The aim of this study is to evaluate the diagnostic accuracy of lung ultrasound for prediction of delayed pneumothorax on chest X‐ray.

**Methods:**

This prospective pilot study was performed between April 2020 and July 2020 in Chungnam National University Hospital. The participants underwent chest X‐rays and lung ultrasound before, immediately after, and 3 h after transthoracic needle biopsy, respectively. The presence or absence of lung sliding at each anterior BLUE‐point on an ultrasound and pneumothorax on a chest X‐ray was recorded.

**Results:**

Pneumothorax occurred in 17 (35.4%) participants, and three of them underwent chest tube replacement. Of the 17 (35.4%) cases of pneumothorax, five participants (10.4%) were diagnosed with delayed pneumothorax. Three out of five participants showed loss of lung sliding on lung ultrasound before the diagnosis of delayed pneumothorax. Therefore, the sensitivity of lung sliding on lung ultrasound for early detection of delayed pneumothorax was 60%. Two undetected cases were asymptomatic, and the pneumothoraces were exceedingly small and recovered spontaneously. Thus, sensitivity for detection of clinically meaningful delayed pneumothorax requiring chest tube replacement was 100% (2/2).

**Conclusion:**

Lung ultrasound can probably predict clinically meaningful delayed pneumothorax after transthoracic needle lung biopsy.

## Graphical abstract

Lung ultrasound is a highly accurate tool for detection of pneumothorax. The aim of this study is to evaluate the diagnostic accuracy of lung ultrasound for prediction of delayed pneumothorax on chest X‐ray after transthoracic needle lung biopsy. The loss of lung sliding after the procedure predicted clinically significant delayed pneumothorax requiring CTD replacement with 100% sensitivity (2/2).

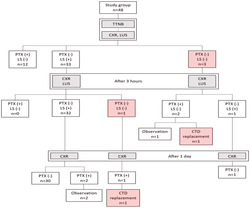

## 1 INTRODUCTION

Transthoracic needle biopsy (TTNB) is a well‐established lung biopsy method, and computed tomography (CT) is the most common modality used to guide the biopsy needle to the target lesion. In addition to conventional CT‐guided TTNB, many guidance modalities have been developed, and recently, the novel electromagnetic guidance system has become available. However, due to the nature of TTNB, a biopsy needle must puncture the pleura, pneumothorax is inevitable. Pneumothorax is the most common complication of TTNB and occurs in about 20%.^1^

Although CT is the gold standard for diagnosis of pneumothorax, it is not frequently used due to cost, radiation exposure, and diagnostic delay. A chest X‐ray is the most commonly used modality for the diagnosis of pneumothorax, but sensitivity is only 46%–52%.^2^ Lung ultrasound is a highly accurate tool for detection of pneumothorax, and in meta‐analyses, its sensitivity and specificity were 87%–88% and 99%–100%, respectively.^2^, ^3^, ^4^

Postprocedural pneumothorax usually occurs within 30 min after TTNB but may occur hours or days after the procedure.^5^ This is called delayed pneumothorax and is defined as the detection of pneumothorax more than 3–4 h after the procedure. The incidence of delayed pneumothorax is 1.4%–4.5%, but it can cause an emergency room visit after discharge and be life‐threatening in case of tension pneumothorax.^5^, ^6^, ^7^, ^8^ Therefore, if possible, an early prediction of clinically significant delayed pneumothorax could be important for safe discharge when the biopsy is performed on an outpatient basis. There have been several studies that have reported risk factors for delayed pneumothorax after TTNB, but no studies have been conducted to establish diagnostic strategies except for chest X‐ray follow‐up.^6^, ^7^

We hypothesized that most delayed pneumothoraces are diagnosed by later enlargement of occult pneumothorax due to the low diagnostic accuracy of a chest X‐ray rather than a new occurrence. Lichtenstein et al. reported “loss of lung sliding sign” on lung ultrasound alone had a sensitivity of 100% for the diagnosis of occult pneumothorax that was not detected in a chest X‐ray.^9^

In this study, we evaluated whether loss of lung sliding that existed before TTNB can predict the occurrence of delayed pneumothorax on a chest X‐ray.

## 2 MATERIALS AND METHODS

### 2.1 Study population

This study was approved by Chungnam National University Hospital Institutional Review Board (CNUH 2019‐02‐031). Between April 2020 and July 2020, 99 patients underwent CT‐guided TTNB (*n* = 74) or EM (electromagnetic)‐guided TTNB (*n* = 25) in Chungnam National University Hospital. The inclusion criteria were as follows: (1) participants with pulmonary lesions who underwent TTNB, (2) 19 years old or older, (3) signed informed consent, and (4) participants who were able to breathe coordinately according to the operator's instruction. The exclusion criteria were as follows: (1) under 19 years old; (2) non‐pulmonary parenchymal lesion (e.g., chest wall, pleural, & mediastinal lesion); (3) participants who were unable to breathe coordinately according to the operator's instruction; (4) pregnant women; (5) there was no normal lung sliding sign on lung ultrasound at either the upper and lower anterior BLUE‐points; (6) appreciate ultrasound images could not be obtained for any reasons prior to TTNB (e.g., severe pleural adhesion); and (7) withdrawal of participation.

### 2.2 Study design and protocol

This is a prospective single‐center interventional study. The participants underwent a chest X‐ray and lung ultrasound prior to TTNB. The presence or absence of lung sliding at each anterior BLUE‐point was recorded, and if lung sliding was not observed in either of the BLUE points, the participant was excluded from the study. The participants included in the study group underwent lung ultrasound and a chest X‐ray immediately after TTNB, and the presence of lung sliding on lung ultrasound and pneumothorax on a chest X‐ray were recorded. Then, participants who had no pneumothorax on a chest X‐ray immediately after the procedure underwent the second lung ultrasound and chest X‐ray 3 h after TTNB. And the third chest X‐ray was performed the day after the procedure.

### 2.3 TTNB procedures

All CT‐guided TTNB procedures were performed according to a standard protocol under CT (Siemens SOMATOM Sensation 64 CT scanner; Siemens Medical Solutions, Forchheim, Germany) guidance by DI Park with 7 years of experience using a coaxial technique (Stericut semi‐automatic cutting needle, TSK Laboratory, Tochigi, Japan). All EM‐guided TTNB procedures were performed using Veran SPiN Perc system (Veran Medical, St Louis, MO, USA) by the same operator (detailed protocols are available in the supporting information).

### 2.4 Lung ultrasound and diagnosis of pneumothorax

**FIGURE 1:**
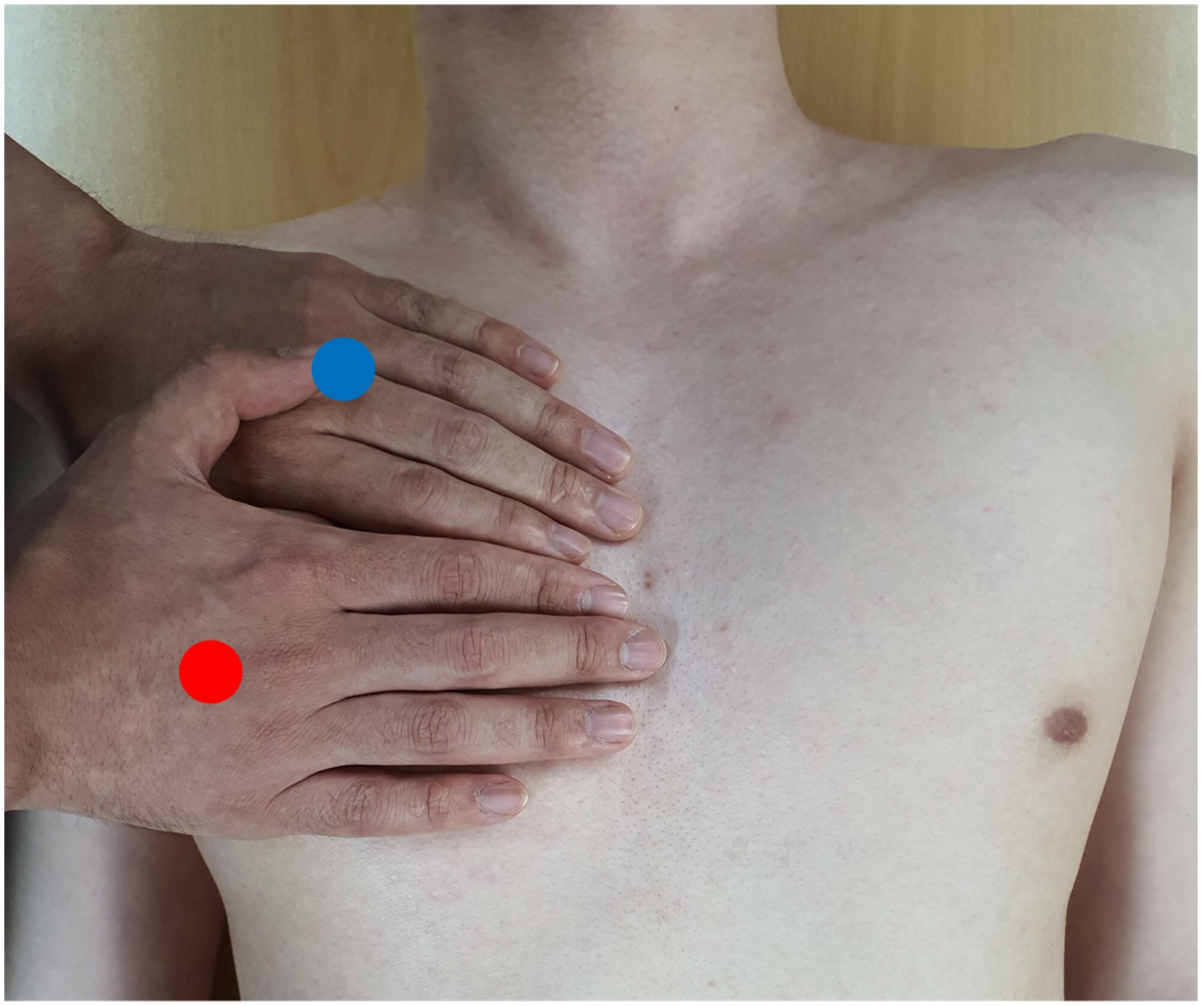
Anterior BLUE‐points (A) in this picture, the lateral side of the upper hand is located on the right clavicle. The upper BLUE‐point is at the middle of the upper hand. The blue dot indicates the upper BLUE‐point. (B) When the side of the right hand excluding the thumb is in contact with the side of the left hand, the center of the palm of the lower hand becomes the lower BLUE‐point. The red dot indicates the lower BLUE‐point

Because ultrasound cannot pass through the air, it is not possible to observe the lung parenchyma directly, but the pleural line, which appears as a hyperechoic line between the ribs, is well observed. The pleurae consist of a visceral pleura, a parietal pleura, and thin space (pleural cavity) containing a small amount of pleural effusion (few milliliters in a normal human) and appear as a subtle sparkling along the pleura on lung ultrasound (lung sliding sign). Lung sliding means that these layers are attached together, so when a pneumothorax occurs, it disappears. In this study, we used a portable ultrasound (Lumify C5‐2, 5 to 2 MHz curved array transducer ultrasound probe, Philips) with a tablet computer (Galaxy Tab S5, Samsung Electronics) and the dedicated application software (Lumify Ultrasound App, Phillips) to detect lung sliding. Lung ultrasound was performed in the intercostal spaces at two standardized points (upper BLUE‐point and lower BLUE‐point).^10^ These two points are located on the anterior chest wall and defined as follows: the upper BLUE‐point is located at the center of the upper hand, and the lower BLUE‐point is located at the center of the lower palm when both hands are placed as shown in Figure 1. Because most pneumothoraces locate anteriorly in supine position, it can be detected at the BLUE‐points with lung ultrasound.

### 2.5 Statistical analysis

Statistical analyses were performed using SPSS Statistics version 22.0 (IBM Corp., Armonk, NY, USA). Continuously distributed variables were expressed as the mean ± standard deviation. Categorical variables were presented as counts and percentages.

## 3 RESULTS

### 3.1 Study group

**FIGURE 2:**
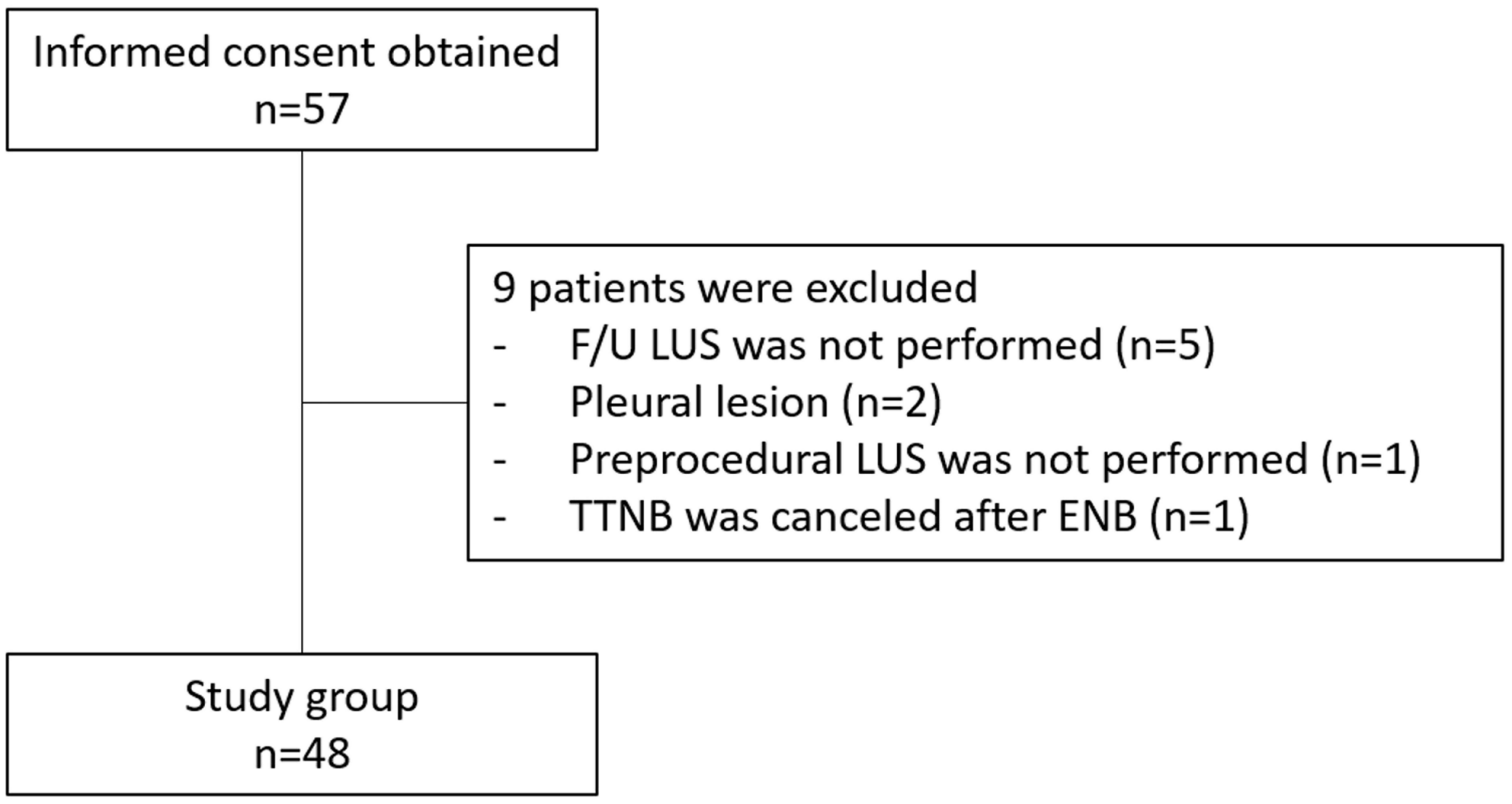
Patient diagram. ENB, electromagnetic navigation bronchoscopy; F/U, follow‐up; LUS, lung ultrasound; TTNB, transthoracic needle biopsy

Of the 57 participants who signed written consent, nine were excluded from the study, and the reasons are as follows: (1) five participants did not undergo postprocedural lung ultrasound, and one of them was excluded because it was impossible to maintain supine position for lung ultrasound due to hemoptysis with dyspnea; (2) two participants had pleural lesions, and normal lung sliding was not observed on preprocedural ultrasound; (3) in one case, EM‐guided TTNB was canceled because the adequate specimens were obtained by electromagnetic navigation bronchoscopy; and (4) we accidentally missed performing preprocedural lung ultrasound in one patient (Figure 2).

### 3.2 Patient, target lesion, and procedure characteristics

Patient, target lesion, and procedure characteristics were shown in Table 1. The mean age of the participants was 69.1 ± 11.6, and males comprised 66.7% (32/48). Among the participants, 28% were ever smokers, and the average smoking amount was 38.6 ± 21.0 pack‐years. The result of pulmonary function test (mean ± standard deviation) showed FEV1 107.0 ± 21.2 (% predicted), FVC 101.2 ± 16.4 (% predicted), FEV1/FVC (%) 72.8 ± 10.8 and DLCO 109.8 ± 19.7 (% predicted). The average size of the target lesion was 43.4 ± 21.9 mm, and 68.7% (33/48) were located in the upper or middle lobe. Of a total of 48 participants, 31 underwent CT‐guided TTNB, and 17 underwent EM‐guided TTNB.

**TABLE 1:** Patient, target lesion, and procedure characteristics (*n* = 48)

Variables	Mean ± standard deviation or number of patients (%)
Age (years)	69.1 ± 11.6
Gender	
Male	32 (66.7)
Female	16 (33.3)
Smoking status	
Ever smoker	28 (58.3)
Smoking amount (pack‐years)	38.6 ± 21.0
Pulmonary function test	
FEV1 (% predicted)	107.0 ± 21.2
FVC (% predicted)	101.2 ± 16.4
FEV1/FVC (%)	72.8 ± 10.8
DLCO (% predicted)	109.8 ± 19.7
Target lesion size (mm)	43.4 ± 21.9
Target location	
Upper or middle lobe	33 (68.7)
Lower lobe	15 (31.3)
Patients position	
Supine	32 (66.7)
Prone	16 (33.3)
Guidance modality	
CT guidance	31 (64.6)
Electromagnetic guidance	17 (35.4)

Abbreviations: CT, computed tomography; DLCO, diffusing capacity for carbon monoxide; FEV1, pretreatment forced expiratory volume in 1 s; FVC, forced vital capacity; NTM, nontuberculous mycobacteria.

### 3.3 Diagnoses

Of the 48 participants, 34 (70.8%) were diagnosed with lung cancer, of which adenocarcinoma accounted for the largest proportion with 25 (52.1%). Among benign diseases, nonspecific benign lesion or inflammation was the most common (*n* = 6), followed by tuberculosis (*n* = 3), nontuberculous mycobacteria (*n* = 2), and parasite infection (*n* = 1). There were two indeterminate cases in which the follow‐up visit was not made (*n* = 1) or the follow‐up duration was not sufficient to confirm the diagnosis (Table 2).

**TABLE 2:** Final diagnosis (*n* = 48)

Diagnoses	Number of patients (%)
Lung cancer	34 (70.8)
Adenocarcinoma	25 (52.1)
Squamous cell carcinoma	3 (6.3)
NSCLC, NOS	3 (6.3)
Small cell carcinoma	3 (6.3)
Tuberculosis	3 (6.3)
NTM	2 (4.2)
Parasite infection	1 (2.1)
Nonspecific benign lesion or inflammation (confirmed by radiology or clinician)	6 (12.5)
Indeterminate	2 (4.2)

Abbreviations: NOS, not otherwise specified; NSCLC, non‐small cell lung cancer; NTM, nontuberculous mycobacteria.

### 3.4 Complications

The overall complication rate in this study was 45.8% (22/48). Pneumothorax occurred in 17 (35.4%) participants, and three of them underwent chest tube replacement. Nine participants showed mild hemoptysis, and all of them improved spontaneously. One of the participants who signed the informed consent showed severe hemoptysis after the procedure and was excluded from the study group because lung ultrasound could not be performed due to the decubitus position for hemostasis. There was no complication related to lung ultrasound (Table 3).

**TABLE 3:** Transthoracic needle biopsy‐related complications (*n* = 48)

Complications	Number of patients (%)
Overall	22 (45.8)
Pneumothorax	17 (35.4)
Observation (oxygen supplement)	14 (29.2)
Chest tube replacement	3 (6.3)
Delayed pneumothorax	5 (10.4)
Observation	3 (6.3)
Chest tube replacement	2 (4.2)
Hemoptysis	9 (18.8)

### 3.5 Prediction of delayed pneumothorax on a chest X‐ray

**FIGURE 3:**
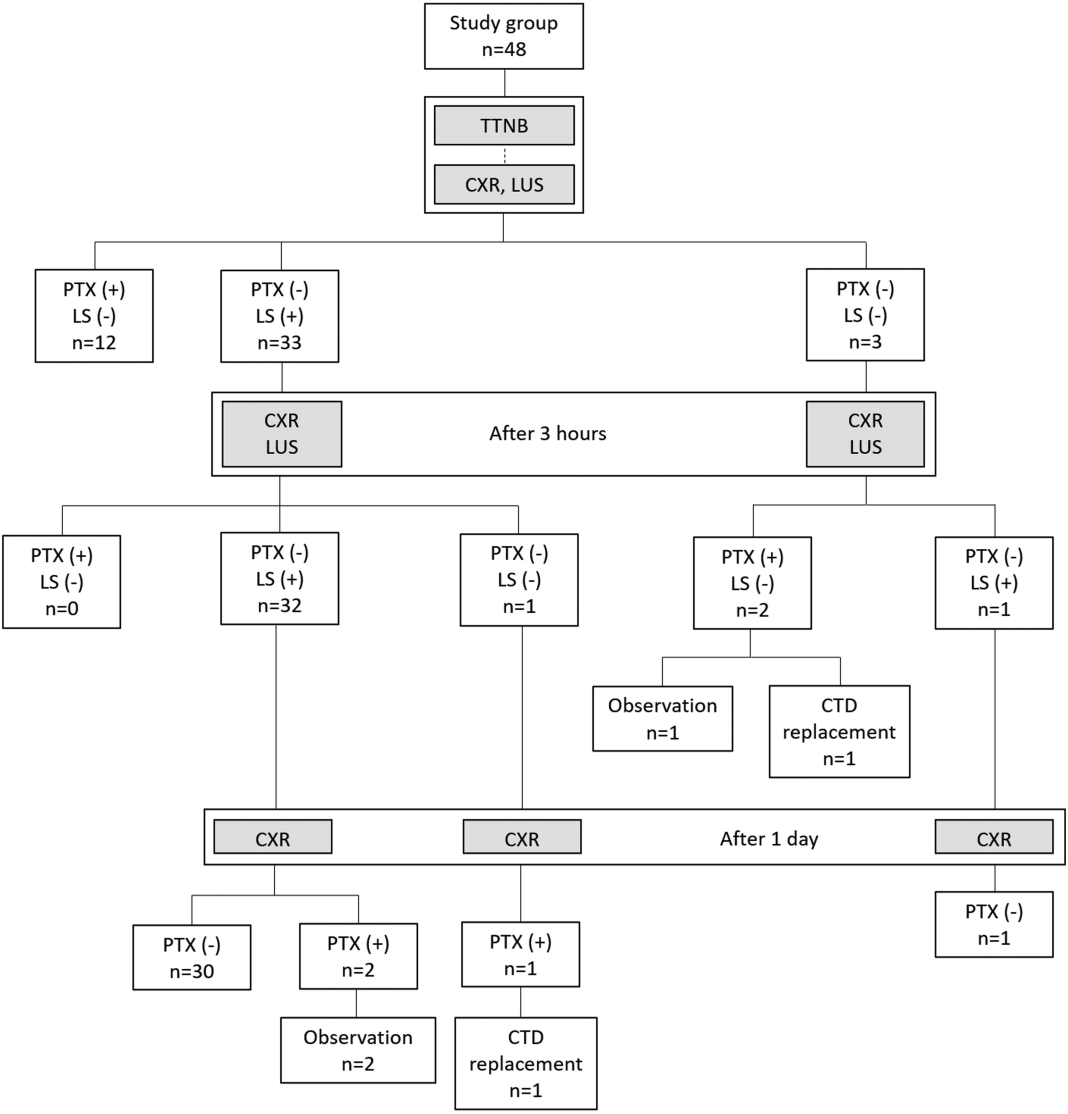
Study flow diagram. CTD, chest tube drainage; CXR, chest X‐ray; LS, lung sliding; LUS, lung ultrasound; PTX, pneumothorax; TTNB, transthoracic needle biopsy

Of the 17 (35.4%) cases of pneumothorax, five participants (10.4%) were diagnosed with delayed pneumothorax. Three out of five participants showed loss of lung sliding on lung ultrasound before the diagnosis of delayed pneumothorax (Figure 3). Therefore, the sensitivity of lung sliding on lung ultrasound for early prediction of delayed pneumothorax was 60% (3/5). Two of the delayed pneumothorax participants who underwent chest tube replacement showed loss of lung sliding before pneumothorax development. The undetected cases were asymptomatic, and the pneumothoraces were exceedingly small and recovered spontaneously. Thus, sensitivity for clinically significant delayed pneumothorax requiring chest tube replacement was 100% (2/2).

## 4 DISCUSSION

Image‐guided TTNB has the widest indication among the lung biopsy methods because it can approach the target lesion regardless of lesion‐bronchus relationship. On the other hand, the risk of pneumothorax is relatively higher due to pleural penetration. After TTNB, the incidence of pneumothorax is an average of around 20%, but it has been reported varying from 9 to 54%.^11^ In the present study, the incidence of pneumothorax was 35.4% (17/48), which was higher than the average in other studies.

One of the reasons for the higher incidence of pneumothorax seems to be the lack of experience in EM‐guided TTNB. The initial EM‐guided TTNB cases (including the first case) conducted in our hospital were included in the study. Among EM‐guided TTNB cases (*n* = 17), pneumothorax occurred in 10 participants (10/17, 58.8%), and three participants underwent chest tube replacement. Pneumothorax occurred in seven participants (7/31, 22.6%) among CT‐guided TTNB cases (*n* = 31), and all of them recovered spontaneously with oxygen therapy. Another reason may be related to a meticulous review of chest X‐rays. Immediately after TTNB, lung ultrasound was performed, and if an abnormal sign (e.g., lung point) suggesting a pneumothorax was present or a normal sign such as lung sliding was loss, chest X‐rays may have been reviewed more rigorously. Therefore, it is possible that exceedingly small pneumothoraces were detected. In fact, one of the undetected delayed pneumothoraces cases was reported as no abnormality in the radiology result.

In this study, delayed pneumothorax occurred in 10.4% (5/48), which was higher than 1.4%–4.5% in previous studies. The absence of emphysema has been reported as a risk factor for delayed pneumothorax. The lack of elastic recoil of the emphysema may prevent the rapid closure of the puncture site and cause early enlargement of pneumothorax.^6^, ^7^ In this study, only 14.6% (7/48) of the study participants had emphysema in the needle pathway, and the pulmonary function tests showed FEV1 107.0 ± 21.2 (% predicted), FEV1/FVC (%) 72.8 ± 10.8, DLCO, and 109.8 ± 19.7 (% predicted), which were slightly better than normal. Therefore, it is highly likely that the participants with no or mild emphysema were included. In addition, while participating in the study, rigorous follow‐up evaluations were conducted during hospitalization in order to detect delayed pneumothorax, whereas in most other studies reporting the incidence of delayed pneumothorax, patients were discharged on the day of the procedure. Thus, the most asymptomatic patients probably made the follow‐up visit after spontaneous resolution.

Lung ultrasound is more accurate than a chest X‐ray in pneumothorax diagnosis and is faster and cheaper than CT. Also, point‐of‐care is possible, and there is no radiation exposure, so it can be repeatedly performed. On the other hand, the sensitivity is 87%–88% lower than that of CT, which can lead to false negative results.^2^, ^3^, ^4^ Most pneumothorax studies using lung ultrasound have been performed in a traumatic setting, so diagnostic accuracy tends to be undervalued. Studies conducted in a non‐traumatic setting showed slightly improved sensitivity of 90%.^2^ Furthermore, Lichtenstein et al. reported that the abolition of lung sliding alone had a sensitivity of 100% and a specificity of 78% for the diagnosis of occult pneumothoraces those were not observed on chest X‐rays.^9^ In this study, participants who were stable in a non‐traumatic setting were included, and the participants who did not have lung sliding on ultrasound were excluded.

In the present study, three out of five participants diagnosed with delayed pneumothorax on chest X‐rays were detected early on lung ultrasound, and the sensitivity was 60%. In two false negative cases, the pneumothoraces were exceedingly small and were observed only in the lung apex. Two cases requiring chest tube replacement were predicted successfully. Therefore, the sensitivity for detection of clinically meaningful delayed pneumothorax requiring chest tube replacement was 100%.

If the patient is hospitalized, delayed pneumothorax does not usually become a serious problem, but if the procedure is performed in an outpatient setting, delayed pneumothorax can cause an emergency room visit or be fatal in case of tension pneumothorax.^5^, ^12^ Using the results of this study, if lung sliding is not abolished 3 h after the procedure, discharge can be considered because the likelihood of clinically meaningful delayed pneumothorax is low.

One of the limitations of this study is that it is a small, single‐center study including 48 participants. Another limitation is that ultrasound was performed only at the anterior BLUE‐points to determine whether lung sliding was abolished. If the size of the pneumothorax is small or there is a pleural adhesion, the location may not be typical. In order to minimize misdiagnosis, it should be considered to perform lung ultrasound at lateral BLUE‐points.

In conclusion, lung ultrasound can probably predict clinically meaningful delayed pneumothorax on chest X‐ray after transthoracic needle lung biopsy. A large multicenter study is warranted to replicate our results for clinical application.

## CONFLICT OF INTEREST

The authors have no conflicts of interest to declare.

## ETHICS STATEMENT

This study was approved by Chungnam National University Hospital Institutional Review Board (CNUH 2019‐02‐031). All participants signed informed consent prior to enrollment in the study.

## AUTHOR CONTRIBUTIONS

JSK, CUC, and DIP contributed to protocol development, data analysis, and wrote the manuscript. JSK, CC, DIP, JOK, SSJ, HSP, JEL, DHK, and KYJ contributed to participant enrollment and management. JSK and CUC contributed equally to the study as co‐first authors. All authors reviewed the manuscript and approved the final version of the manuscript.

## Supporting information


**Data S1.** Supporting information

## Data Availability

The data that support the findings of this study are available from the corresponding author upon reasonable request.
