# Supplementary material for: Early detection of delayed pneumothorax using lung ultrasound after transthoracic needle lung biopsy: A prospective pilot study
Source: Clin Respir J. 2022 May 20;16(5):413–9. doi: 10.1111/crj.13495 (PMC9366587; doi:10.1111/crj.13495)
Supplement: Supplementary file 1 — Data S1. Supporting information [file CRJ-16-413-s001.docx]

**Detailed procedure protocols**

1. **CT-guided TTNB procedure**

All CT-guided TTNB procedures were performed under CT (Siemens SOMATOM Sensation 64 CT scanner; Siemens Medical Solutions, Forchheim, Germany) guidance by DI Park with 7 years of experience in CT-guided TTNB. CT-guided TTNB procedure was conducted in the following order: (1) prior to CT scan, the participants were instructed to breath in tidal volume and hold it according to the operator’s instructions during the procedure; (2) After obtaining CT scout image with a radiopaque grid attached, a low-dose (100 kVp, 20 mA) CT scan was performed with 2 mm slice thickness; (3) the skin entry point was determined using CT images and marked using a skin marker; (4) the entry point was sterilized using povidone-iodine 10% solution; (5) 2% lidocaine solution was administered from the skin to the parietal pleura for local anesthesia; (6) a coaxial introducer needle was introduced through the skin entry point; (7) CT scan was performed to ensure that the needle tip was in target lesion; (8) Stericut semi-automatic cutting needle (TSK Laboratory, Tochigi, Japan) was inserted via the coaxial introducer needle, and two to three biopsies were performed.

1. **EM-guided TTNB procedure**

All EM-guided TTNB procedures were performed using Veran SPiN Perc system (Veran Medical, St Louis, MO, USA) by the same operator (DI Park). EM-guided TTNB procedure was conducted in the following order: (1) all participants underwent chest CT scan with 0.7 mm slice thickness to create a virtual three-dimensional (3D) map for navigation on the day of the procedure or one day before; (2) the target lesion was marked and the pathway to the target lesion was made on the virtual 3D map, the skin entry site was also determined for in case of EM-guided TTNB using a dedicated software (SPiN Drive System) (planning phase); (3) electromagnetic navigation bronchoscopy was performed using a 4.0 mm thin bronchoscope (BF-P260F, Olympus) and the dedicated instruments (forceps or needles) (navigation phase); (4) if the navigation succeeded, transbronchial lung biopsy was performed; (5) if the navigation was not successful or failed to obtain adequate specimens, then, EM-guided TTNB procedure was followed in the same bronchoscopy suite; (6) the entry point was sterilized using povidone-iodine 10% solution; (7) 2% lidocaine solution was administered from the skin to the parietal pleura for local anesthesia; (8) a coaxial introducer needle (Always-On Tip Tracked® biopsy needle, Veran Medical) was introduced through the skin entry point under real-time virtual guidance (figure 1); (9) Stericut semi-automatic cutting needle (TSK Laboratory, Tochigi, Japan) was inserted via the coaxial introducer needle, and two to three biopsies were performed. All procedures were performed under moderate or deep conscious sedation.

Since most pneumothoraces locate anteriorly in supine position, it can be detected at the BLUE-points with lung ultrasound.
